# Who are CHWs? An ethnographic study of the multiple identities of community health workers in three rural Districts in Tanzania

**DOI:** 10.1186/s12913-019-4563-6

**Published:** 2019-10-21

**Authors:** Mohamed Yunus Rafiq, Hannah Wheatley, Hildegalda P. Mushi, Colin Baynes

**Affiliations:** 1 Ifakara Health Institute, P.O. Box 78373, Dar es Salaam, Tanzania; 2 P.O Box 337, Bagamoyo, Tanzania

**Keywords:** Community health workers, Multiple identities, Family planning, Ethnographic, Curative, Preventative, Referral and intermediary, Tanzania

## Abstract

**Background:**

Numerous studies have examined the role of community health workers (CHWs) in improving the delivery of health services and accelerating progress towards national and international development goals. A limited but growing body of studies have also explored the interactions between CHWs’ personal, communal and professional identities and the implications of these for their profession. CHWs possess multiple, overlapping roles and identities, which makes them effective primary health care providers when properly supported with adequate resources, but it also limits their ability to implement interventions that only target certain members of their community, follow standard business working days and hours. In some situations, it even prevents them from performing certain duties when it comes to sensitive topics such as family planning.

**Methods:**

To understand the multiple identities of CHWs, a mixture of qualitative and ethnographic methods was utilized, such as participant observation, open-ended and semi-structured interviews, and focus group discussions with CHWs, their supervisors, and their clients. The observation period began in October 2013 and ended in June 2014. This study was based on implementation research conducted by the Connect Project in Rufiji, Ulanga and Kilombero Districts in Tanzania and aimed to understand the role of CHWs in the provision of maternal and child health services in rural areas.

**Results:**

To our knowledge, this was the first study that employed an ethnographic approach to examine the relationship between personal, communal and professional identities, and its implications for CHWs’ work in Tanzania. Our findings suggest that it is difficult to distinguish between personal and professional identities among CHWs in rural areas. Important aspects of CHW services such as personalization, access, and equity of health services were influenced by CHWs’ position as local agents. However, the study also found that their personal identity sometimes inhibited CHWs in speaking about issues related to family planning and sexual health. Being local, CHWs were viewed according to the social norms of the area that consider the gender and age of each worker, which tended to constrain their work in family planning and other areas. Furthermore, the communities welcomed and valued CHWs when they had curative medicines; however, when medical stocks were delayed, the community viewed the CHWs with suspicion and disinterest. Community members who received curative services from CHWs also tended to become more receptive to their preventative health care work.

**Conclusion:**

Although CHWs’ multiple roles constrained certain aspects of their work in line with prevalent social norms, overall, the multiple roles they fulfilled had a positive effect by keeping CHWs embedded in their community and earned them trust from community members, which enhanced their ability to provide personalized, equitable and relevant services. However, CHWs needed a support system that included functional supply chains, supervision, and community support to help them retain their role as health care providers and enabled them to provide curative, preventative, and referral services.

## Background: community health workers

Over the past two decades, official and scholarly discourse has viewed CWHs as a panacea for a range of vertical and health system challenges [1–5]. A systematic review conducted by the World Health Organization (WHO) using eight in-depth country cases suggests that CHWs can potentially improve population level health through provision of maternal and child health services, case management of uncomplicated illnesses, and by engaging in preventive education on malaria, Tuberculosis (TB), HIV/AIDS and Non-Communicable Diseases (NCDs) [4, 6]. CHWs in South Africa have increased access to HIV/AIDS services and counselling by making them a focus in both health care centers and during homestead visits [3]. However, as Uta Lehmann and David Sanders suggest in the WHO report titled, “Community Health Workers: What do we know about them?”, for CHWs to continue being effective in improving maternal and child health, they need to be carefully selected, trained, supervised, and supported [1]. Community workers, as the authors suggest, need to be seen as part of the social and health system, “in which different actors are linked with each other in chains of relationships” [7, 8].

CHW programs continue to gain prominence with the increase of infectious and non-communicable diseases in urban contexts of developing countries. CHWs have been used in Kenya to address “neglected tropical diseases” such as schistosomiasis in informal urban settlements [9]. Researchers in Kenya noted that due to CHWs’ local status, community members were more willing to participate in the research. CHWs also provided extensive information about the areas they worked in, which informed the treatment plan for the schistosomiasis project in Kenya [9]. Similarly, in the wake of the Ebola outbreak in Sierra Leone, the United Nations Population Fund (UNFPA) and government trained CHWs practiced “contact tracing,” a method of “tracking contacts, or people linked to confirmed or probable Ebola cases” [10]. Tapping into the close-knit infrastructure of local CHW networks, this method assisted in the early detection and rapid treatment of Ebola and has become a model for disease surveillance in different African countries [10].

To address health systems challenges, improve formal health services utilization, and to target specific health challenges such as infectious and non-communicable diseases, program designers and countries have designed and experimented with different types of CHWs programs [2, 4, 11]. CHW programs have generally been divided into generalists and specialist types. Generalist CWHs have mostly been volunteers used both by government and non-governmental programs to provide curative and educational services and assist in vaccination campaigns [1, 2, 12]. Specialist CHWs often focus on specific areas such as tuberculosis care and malaria control [1, 13]. The level of training and areas of focus between CHW programs also vary [12]. Some CHWs undergo a short training, typically lasting two weeks, and work in basic preventive and curative services [4]. Others are trained for an extensive period of time and provide a wide range of preventive and curative services [2, 4]. Some are compensated for their work, have a strong clinical focus, and provide verbal and assisted referrals, while others work as unpaid volunteers [1].

In 2014, the Tanzanian Ministry of Health approved a community-based health program (CBHP) policy guideline that called for a special type of a CHWs. This policy envisioned a special cadre of CHWs who would be locally selected and deployed. They would be trained based on a government approved curriculum and employed by the government. These CHWs would provide an integrated and comprehensive package of services including Reproductive, Maternal, Newborn, Child and Adolescent Health (RMNCAH), connect households to facility services, engage in preventive and curative services, and provide disease surveillance [14, 15]. While there is a rich body of literature on the vital role that CHWs play in health services delivery, there is a limited amount of literature on how CHWs’ personal and communal identities interact with their current professional roles and the implications this has on their work. CHWs in sub-Saharan Africa typically work in rural settings, where personal and professional roles are not easily differentiated. Often, these roles are performed simultaneously blurring the lines between the domestic and professional work [16–18].

The current study builds on a limited but growing body of literature on the identity of CHWs—who are they?—and their intermediary role linking the community to the health care system [1, 18]. Several recent studies noted that CHWs’ personal and communal roles and identities interact with professional roles enhancing and sometimes impeding their health services delivery work [7, 17, 18]. In their study on rural South Africa, Mlotshwa et al. argued that CHWs’ existing identities, as a community members or farmers, played an important role in their recruitment as CHWs, the services they delivered, and the level of trust they developed with their patients [18]. In two studies dated 2015 and 2017 by Kok et al., the authors also confirmed these findings. The authors observed in their transition into a professional role as a health care worker, CHW’s “insider role” was positively evaluated by community members, who saw one of their “own” bringing in services and opportunities that did not exist before [7, 18, 19]. However, CHWs professional role created tensions and frictions with the community. In some occasions, the CHWs were viewed with suspicion and seen as gatekeepers to vital external resources [7, 18, 19].

Similarly, in a study by Mumtaz et al. the authors listed many barriers that faced lady health workers (LHW) in Pakistan. The authors noted that LHW faced conflicts in balancing their domestic and professional work [17, 20]. Domestic work such as collecting water or firewood, despite enhancing social relations and building trust among villagers, was not associated with the delivery of professional services. In Kok et al. study, the authors also showed that CHWs experienced tension and stress from their new roles because it created different expectations [7, 19]. For the community, CHWs represented prospect for more curative services. Politicians used the CHWs for their various political campaigns and meetings while health care workers expected that CHWs would advise more community members to attend formal health care centers [7, 19].

Other studies have examined CHWs’ use of time indirectly suggesting that the “official time” may mask other uses of time influenced by local identities and roles. In Tani et al. study in Tanzania, the authors examined CHWs’ use of time during their 8:00 AM to 4:00 PM work regimen, breaking down the type of services they provided and amount of time spent on each activity [21]. However, the authors noted that while their study provided a useful picture of how CHWs spent their official time, it did not capture services and activities that were provided beyond non-official time. In this regard, CHW’s jobs do not fit neatly into the standard 07:30 AM to 5:30 PM work schedule of the Tanzanian government. CHWs may be called on to serve their communities at all times of day and night, at any place they meet the client. Like Mlotshwa et al. study, CHWs in Kilombero assisted in the escorting of pregnant mothers on their way to delivery, attended to accidents, bought supplies on behalf of their patients and re-visited patients for checkups after official work hours [21]. These “non-professional” activities are not captured in the official time use and seem to ignore the role of pre-existing identities in motivating CHWs to provide services during off hours [18].

## Background: connect project in Tanzania

In 2010, Connect, a project conducted under Ifakara Health Institute (IHI), introduced a full-time paid CHWs intervention program in three Tanzanian districts: Kilombero, Ulanga and Rufiji. Connect’s unique cadre of health workers were called community health agents. In Swahili, they were known as as *Wawezeshaji wa Afya ya Jamii* (WAJA). They were called agents (as opposed to “workers”) to emphasize their role in facilitating change towards healthy life and connections between the community and the formal health sector. Tanzania’s Ministry of Health and Social Welfare’s 2007–2010 Primary Health Services Development Program and the fourth Health Sector Strategic Plan of 2015–2020 both called for multi-purpose CHWs with standard training and a remuneration package [5, 22]. Connect’s aim was to test the Ministry of Health’s hypothesis that the deployment of paid, well-trained, multi-purpose CHWs with adequate health system support would accelerate the attainment of Millennium Development Goals (MDGs) 4 and 5, which aim to reduce child and maternal mortality [23]. Connect used a community-based clustered randomized controlled trial research design (RCT) to test *Mpango wa Maendeleo wa Afya ya Msingi* (MMAM), Tanzania’s primary health services development program vision. The RCT design included 101 villages, and WAJAs were randomly assigned to 50 intervention sites.

The Connect project was designed to increase greater community involvement in program operations by using residents as WAJAs and local leaders as their supervisors [24]. The criteria for selecting WAJA required the candidates to be village residents, possessing a secondary education, and willing to serve their community [23, 24].

Selection of WAJAs was done openly in a village assembly. A total of 146 WAJAs in three cohorts from 50 villages in Kilombero, Ulanga and Rufiji were trained. Although WAJAs ranged in age from 18 to 45, most were young and were perceived by the community as youth [23]. The villages advertised the position and the community voted to select its WAJAs. Once selected, WAJAs were trained for a period of nine months at IHI’s headquarters in Ifakara, Tanzania and then returned to their home villages. Their support system included three district intervention coordinators. It included also WAJA focal persons nominated by each district to oversee project’s activities and two supervisors at the village level to assist in community and medical advice.

**Fig. 1:**
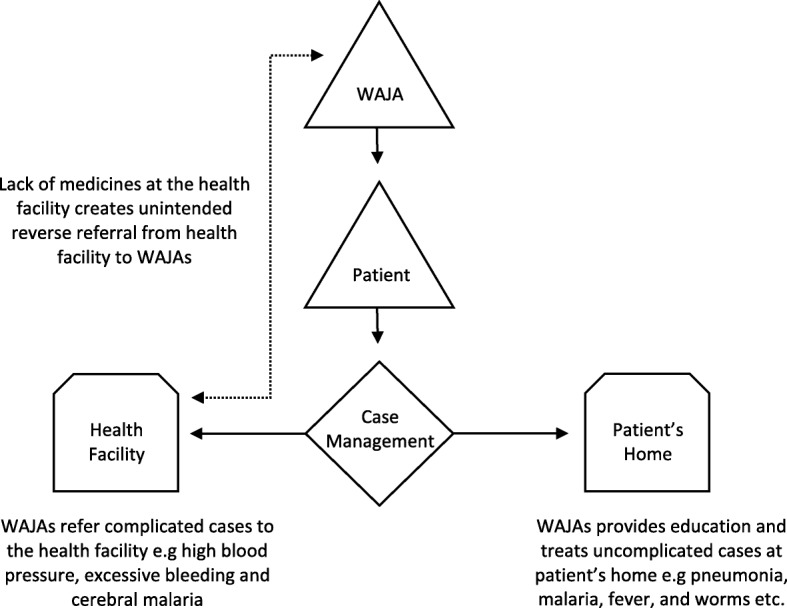
WAJAs play a case management role between the patient and the health facility

WAJAs work included curative services for uncomplicated cases, referrals to formal health centers and preventative health education (See Fig. 1). WAJAs provided a roster of health services, including treatment for children under the age of five for uncomplicated malaria, pneumonia and diarrhea, referral of complicated cases to formal health centers as well as health education on family planning, safe motherhood, essential neonatal care, integrated management of child illness and basic hygiene (See Fig. 1). WAJAs provided these services in their home villages (See Fig. 1). Each village was typically assigned one male and one female WAJAs, however for larger villages in population and area, the number of WAJAs was increased to three WAJAs per village. Each WAJA served an average population of 2000 villagers. Every week, WAJAs created a work schedule that estimated 40 h of work. To ensure quality and the execution of work, the health facility supervisor and village supervisor roles were created to support the WAJAs. The two supervisors were given the WAJAs’ work schedule every week for review and to plan follow-ups. In many instances, the village supervisor accompanied the WAJAs on their household visits. For their services, WAJAs were paid an equivalent of $120 per month in comparison to formal health care workers at the health centers who received $300 per month. The cost of training one WAJA was $1348.21 [25]. Village supervisors were unpaid.

### Study aim and research questions

This study aimed to understand the community’s reception of community health workers in Connect intervention areas using ethnographic approach. We pursued three research question to understand the study’s main aim: (1) How are CHWs professional roles enacted? (2) Do professional roles interact with other CHWs roles? (3) If so, which ones and how? Understanding CHWs existing roles and identities, and how they interact with professional roles is vital in strengthening CHWs recruitment and motivation, program design, and success [18].

### Patient’s journey

#### Conceptual framework

Our conceptual model draws from the theory of multiple identities and open systems thinking [26–29]. We first draw from the theory of multiple identities, which proposes that individuals have multiple overlapping identities which vary overtime and according to specific contexts. Furthermore, these multiple identities, such as being a WAJA and a son or daughter, sometimes exist in tension with each other as groups and individuals make demand on the actors [25, 26, 29]. In return, the individuals negotiated these competing demands by deploying one or several roles and identities over the others [31]. To situate how WAJA, as health providers, are part of a wider social system, we draw from open systems thinking on health [29]. Systems thinking approaches health providers as part of a wider environment, which is not limited to material aspects but include economy, politics, gender norms and formal and informal institutions. These contexts exert force on the health care providers and vice versa. All aspects of the social system impact health care providers and vice versa as none of them work in isolation. While different institutions are separate from one another, it is important to recognize that the boundaries between them are porous and interdependent [29].

### Conceptual model for WAJAs’ multiples identities

**Fig. 2:**
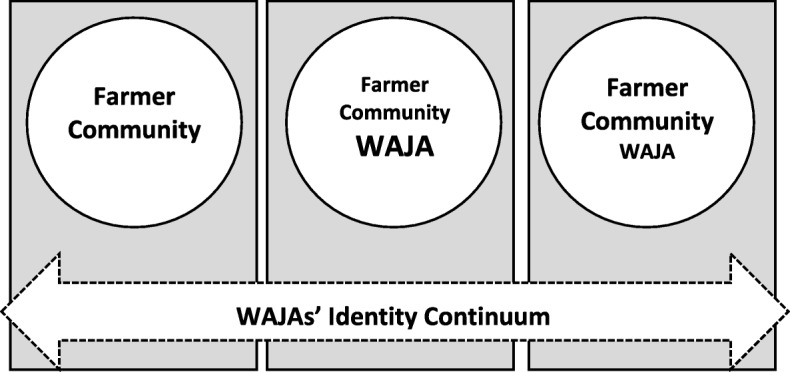
Prior to project entry, WAJAs inhabited several roles including being farmers and community members. With the entry of the project, WAJAs acquired roles as health provider which coexisted but dominated existing roles. During the times when medical and salary were delayed, WAJAs’ professional roles becomes diminished while their roles as farmers and community members become highlighted

Accordingly, we viewed the WAJA’s role in the health care system following some of the ideas and principle espoused in the multiple identity and open system thinking. In our simplified model, see Fig. 2, we show that prior to the project’s arrival WAJAs inhabited various roles such as farmers and community members. We use farmers in our model as a placeholder for a range of “non-professional” money-making activities that WAJA engaged in addition to their salaried position. Although almost all WAJAs farmed their own land or were paid laborers in others’ fields at certain times of the year, many WAJAs also participated in less seasonal side work such as brick making, motorcycle-taxi driver, making palm oil, and fishing. We use community member as a label to signal a wide range of social, spatial and political affiliations such as being a village resident, a healer, religious and ethnic member or belonging to village-level associations such as village government, health committees or a dance group. WAJA identities consisted of both personal, communal and professional roles.

Changes in WAJAs’ roles and identities did not remain static but varied with the changing contexts as our conceptual model shows. In open systems, when one-part changes, such as access to medicine, it influences other parts of the system: community’s evaluation of WAJA’s status as a professional. As the project progressed, WAJA’s professional role became amplified through access to medicine, salary, training and symbolic capital associated with a powerful project. Inversely, their identity as farmers and community members became diminished in comparison to their new role as WAJAs (See the second box in Fig. 2). However, when medical supplies and salaries were delayed, the professional WAJA’s role again diminished. As the third box in Fig. 2 shows, their personal and communal roles became more amplified than their professional roles as WAJA.

## Methods

### Settings and study population

The data for this article comes from research conducted during two related projects in Kilombero district, Morogoro region. The main project was called Connect, a research intervention study designed to test the impact of using a paid cadre of CHWs that provided integrated maternal, newborn and child health service [23]. CHWs also provided family planning services such as distributing condoms, refilling oral contraceptives, and providing education and referrals (for other family planning methods) in households. The second sub-project was known as Connect Family Planning, which began to operate in 2013. It aimed to contextualize the findings in the first project, which had shown that CHWs had a non-significant effect on contraceptive utilization after two years of their introduction [32]. During the implementation of both studies from 2010 to 2013, CHWs retention was 98% (Kante, Almamy. Personal communication. Aug.21, 2019).

The study population comes from rural and peri-urban areas. The residents of Kilombero District are mostly engaged in subsistence farming, cultivating crops like rice, maize, and cassava. Kilombero District is a religiously and ethnically heterogeneous area, populated by both Muslims and Christians [33]. Common ethnic groups include farming tribes such as the Wapogoro, Ndamba, Kaguru, Wangoni, Wahehe as well as recent migrants such as the Sukuma, who are both farmers and pastoralists. At times, conflict would arise between farmers and pastoralists. During the study period, such a conflict led to the death of a police officer, siege of a police station, and having helicopters and other national reinforcements brought to the area [34].

### Data collection and analysis

The data informing this article comes from two sources: (i) qualitative data in the form of interviews (IDIs) and focus discussions (FGDs) and (ii) ethnographic data in the form of observations and participation. The qualitative research was part of a larger study, which was registered through the International Standard Randomized Trial register with an award number ISRCTN96819844. The IDIs and FGDs were collected in two phases during March 2012 and during July 2013 from eight villages out of 50 intervention villages in Rufiji, Ulanga, and Kilombero Districts. Qualitative data collected during March 2012 came to be known as Qualitative Appraisal System 1 (QSA 1), while data collected during July 2013 came to be known as Qualitative Appraisal System 2 (QSA 2). Fewer interviews were conducted during QSA II because the aim was to track any changes in the specific themes rather than producing an exhaustive list of themes. The criteria for selecting the villages for QSA 1 and 2 factored in the size of the villages, numbers of WAJAs deployed, and information about health coverage and access. The aim of the data collection was to gain impressions from different stakeholders and perspectives involved in both the provision and receiving of health services. Researchers found saturation in the targeted themes: improvement of maternal and child health, referrals, medical supplies, and increased knowledge of MCH health in order to triangulate the quantitative data. From the village government, participants included Village Executive Officers (VEOs), village chairmen, traditional birth attendants, Village Health Workers, village supervisor, hamlet leaders and WAJAs. From the government and health care providers, the respondents included health care workers (doctors, clinicians and nurses) and members of the Community Health Management Team (CHMT).

A total of 88 IDIs and 24 FGDs were conducted by native Swahili-speaking interviewers (see Table 1). On average, each interview lasted for forty-five minutes to one hour and the FGD took between sixty and ninety minutes. The FGDs averaged 12 respondents, including women and men, categorized by age, gender and profession [35]. An additional file has been included that shows more details on the questions administered (see Additional file 1). Upon consent, interviews were audio recorded and then translated into English by experienced translators. In both rounds of qualitative data collection, the same interviewers were used for consistency. Prior to data collection, the interviewers were trained by a senior IHI researcher on research ethics and confidentiality as well as how to correctly phrase the interview and focus group guides. Community and district authorities assisted the researchers in identifying respondents from the Connect sites. The researchers had a list of positions in the community as well as categories. The local authorities would introduce the researchers to the appropriate individual occupying the requested position or a representative from the community for the requested categories. A list of the positions and categories of the respondents can be found in Table 1.

**Table 1:** Descriptions of IDIs, FGDs and Types of Informants

	QSA 1 March 2012	QSA 1 March 2012	QSA 2 June/July 2013	QSA 2 June/July 2013
	Kilombero and Ulanga	Rufiji	Kilombero	Rufiji
Key Informant Interview				
Village Executive Officer (Mtendaji)	2	2	3	3
Village Chairperson (Mwenyekiti)	2	2		
Traditional Birth Attendant	2	2		
Village Health Workers (WAVI)	2	2		
WAJA Village Supervisor	2	2	3	3
WAJA	2	2	3	3
Hamlet (Kitongoji) Leader	2	2	3	3
District Medical Officer	1	1	1	1
Medical Officer In-Charge (facility)	1	1		
WAJA Focal Person	1	1	1	1
District Reproductive and Child Health Coordinator	1	1	1	1
District Pharmacist	1	1		
District Health Information Officer	1	1		
District Health Secretary	1	1		
Chair of Council Health Services Board	1	1		
Health Facility in Charge (Head Clinician)	2	2	3	3
Clinical Supervisor of WAJA	2	2		
Subtotal	26	26	18	18
Total IDI	QSAI IDI 52		QSAII IDI 36	
Focus Group Discussions				
Women with child under 1	2	2		
Men with children under 5	2	2		
WAJA	2	2	1	1
Members of Village Government	2	2		
WAJA Village Supervisors	2	2		
WAJA Clinical Supervisors	1	1		
Subtotal	11	11	1	1
TOTAL FGD	QSAI FGD 22		QSAII FGD 4	

The second source of data for this article comes from ethnographic research that involved observation and participation in WAJA’s professional, communal and personal activities. Two researchers were involved in the ethnographic study, a medical anthropologist completing his doctoral degree and a research assistant with a university degree. The ethnographic study occurred over a nine-month period from October 2013 to June 2014. It involved four villages in Kilombero District: Katindiuka, Lumemo, Mlabani and Kisawasawa. Researchers accompanied supervisors distributing supplies to the WAJAs, attended training of village health teams, observed mass meetings of WAJAs and district supervisors, reviewed WAJAs’ monthly reports with the supervisors, visited health centers and dispensaries, interviewed health workers at the centers and dispensaries, and interviewed community members about WAJAs’ services.

Our research also included an observation period that entailed visiting six WAJA, four females and two males, three times a week for 12 weeks in their villages. We spent an average of six hours a day observing and participating in WAJA activities alternating between morning and evenings among the four villages. We kept a daily record of our observations in the form of field notes, and we discussed salient findings and topics at the end of each day. The ethnographic data focused on WAJA’s professional work including household visits, which entails case management, educational sessions on maternal and child health and family planning, referrals, patient check-up and consulting with supervisors and fellow WAJAs over the phone. The data also included WAJA’s “non-professional” money-making roles primarily farming but also brick making *boda boda* (motorcycle) taxi driving and the pressing of palms for oil. The other data was around their communal roles - attending meetings, prayers and community gathering such as funerals, weddings and baptisms.

Prior to beginning the ethnographic research, we analyzed QSA 1 and 2 qualitative data to discover broad themes and topics related to the WAJAs’ reception in their own villages. Three team members were involved in reading the IDI and FGDs including one member who was part of the data collection in QSA 1 and 2 and two members who were not part of the data collection team. The initial analysis of the qualitative followed grounded theory procedure, an inductive research method that strives to generate concept within the data, privileging description over abstract categories and engaging in constant comparison between data sets [36]. We pursued open coding to determine the frequency, similarities, relationships and contexts that shape WAJAs reception in the study area. Based on the general findings, we adopted categories such as lack of medicines, delays of salaries, kinship relations and income generation activities all of which emerged from the reading of IDIs and FGDs. This was achieved through writing memos, sharing notes and conducting discussion among the three researchers. We also recorded salient quotes and information from interviewees based on the general analysis of the IDIs and FGDs data. We used these first impressions, themes and topics to guide us but not to determine the scope of our ethnographic research.

We repeated the same process for our ethnographic data. We pursued open coding to analyze our field notes by constantly reflecting on themes generated earlier from the qualitative data, noting frequency and similarity of themes, levels of detail, and relationships among the IDIs, FGDs and ethnographic fieldnotes. We wrote memos, shared notes and met to discuss to draw out the connections, similarities and different aspects of our data. At the axial coding stage, we engaged in an overall interpretation of data based on the patterns that emerged. This procedure verified earlier observations such as delay of medical supplies but also revealed a richer picture of the implications of such findings to WAJA’s professional role. Axial codes were combined to provide an explanatory framework of how WAJAs’ roles and identities blur and interact with implications to their work. At the final, integrative stage, we generated a working theory to explain how previous and new roles interacted positively and negatively on WAJAs’ work.

### Strengths and limitations of the study

The strength of this study was the use of ethnographic approach to understand community’s reception of the introduction of a specific type of community health workers. Therefore, the researchers spent nine months observing the WAJA and were able to triangulate and contextualize the findings in QSA I and II. To minimize observer’s effect, researchers accompanied WAJAs on their pre-planned work schedules instead of creating activities for the researchers. The limitation of the current study is that the data used was collected five years ago. Several of the issues that undermined CHWs professional and personal identity such as delays of salaries or medical supplies may have changed or resolved. Another weakness is the study’s sample size particularly for observational data. The researcher observed only six WAJAs from four villages in Kilombero district, which means the issues and factors observed during the study were place and time-specific.

## Results

The direct quotes included below come from participants in QSAI, QSAII and the ethnographic study period. We identified four main themes: Economic Activities and Kinship Ties; Merging Kinship Ties with Professional Relationships; Stakeholder Ownership; Access to Medicine as Professional Identity; and WAJAs Local Knowledge and Efforts to Provide Services.

### Pre-existing roles: economic activities and kinship ties

In the ethnographic component of the study, five out the six WAJAs whom we observed were engaged in income generation activities outside official eight-hour per day, five days per week duties as WAJA. These included farming, brick-laying, extraction of palm oil and driving motorcycle taxis. These activities were done during both work and non-work hours. Female WAJAs preferred mostly small business such as palm oil extraction and farming rather than driving motorcycle taxis or brick making, which were socially prescribed as male occupations. WAJAs’ “non-professional” work increased during farming season (December to mid-May) and during the frequent delays in their salaries and medical supplies. The WAJAs informed us that these activities were undertaken to supplement their professional salaries and meet family and societal demands, such as the support of siblings and financing of weddings and funerals. Some of these activities, such as contractual farming (known as the *Mraba* system), were undertaken to supplement their salaries:

*At that time, I had not received my salary yet, honestly I was working as a WAJA for two days, and the rest I had to look for other work; like someone will offer me a piece of land (Mraba) to cultivate, and in the end he will pay me, so that when I go back home I can have a meal, and so that they [WAJA’s family] can see that I am working. It is not like permanent work.* (Field Notes, Male WAJA from Kilombero District)

WAJA interviewed stated that he exchanged his labor for the *Mraba* to supplement his income and fulfill familial obligations. The male WAJA noted that Mraba contracts were common in the planting season that begins in December and extends through late March in Kilombero District.

Working as a contract farmer formed WAJAs personal and communal identity, which also connected them to the socio-economic sector of farming. This in turn enhanced their professional identity as health care workers. During rainy seasons, farmers move into their farms where they spent extended amount of time preparing the rice fields and planting. As farmers, WAJA knew about the state of roads during heavy rains and the difficulty of reaching communities that needed care in areas prone to flooding. During rainy seasons some roads were impassable even by cars. WAJA also acquired knowledge of the kinds of diseases and health challenges that different seasons brought. Farmers lived in the flooded fields and their drinking water at this time came from five-foot pits contaminated by the floods, which acerbated intestinal diseases like cholera and diarrhea. Health care workers also knew about these issues; however, because their services were provided in a fixed position, their knowledge about community norms and disease trends were not as in-depth and timely as that of WAJAs. WAJA used this knowledge to inform their work but also in assisting government-initiated campaigns against cholera and diarrhea.

### Merging of kinship ties with professional relationships

The bonding between WAJA, project staff and the community was evident in the use of kinship terms to refer to each other. Community members, village government officers, health facility employees and Connect project staff members referred to WAJA using kinship terms like *vijana* (youth) *WAJA wetu* (our WAJA), *(m) wanangu* (my offspring) and *watoto* (children). In the following excerpt, a health facility supervisor expresses his positive working relations with his local WAJA:

*In general, we don’t have a problem with our WAJA [WAJA wangu], other [WAJA] can call us asking us if the client they have referred has reached the clinic. And we give him feedback on the situation* (IDI, Male Health facility supervisor from Rufiji District)

Health facility supervisor referred to WAJA using a kinship terms like *WAJA wangu,* an identity label commonly used in personal and community interactions. In turn, WAJA also called some project supervisors Mama WAJA, which means WAJA’s mother. Community members, project staff and WAJA themselves used identity labels that blurred the distinction between personal, community, and professional domains.

WAJAs status as youth and kin members made villagers, especially youth, more comfortable and willing to ask them questions, attend their educational sessions and ask for contraceptives such as condoms. In our household and neighborhood visits with WAJAs, youngsters would regularly stop the WAJAs to ask for condoms and ask questions about sexual health.

### Access to medicine as professional credibility

Interviews with WAJAs and observations of their interactions with clients revealed that when WAJAs had access to medicine they were seen as professionals, but when they do not have medicine they were treated with suspicion. In addition to providing health education, arranging referrals for complicated cerebral malaria cases and high-risk pregnancies, WAJAs also engaged in case management of mild malaria, pneumonia, and diarrhea. WAJAs provided free medication to mothers and children under five, visiting their client door-to-door. At times, WAJAs were able to dispense medicines even when local dispensaries and health clinics lacked the necessary supplies. Community members often refer to WAJAs as “street doctors” and female WAJA as “street nurses” because of their access to medicine. Sometimes, the label “street doctor” was used regardless of WAJA’s gender. In the following passage, a male WAJA from Rufiji explains why they are associated with being doctor-like:

*Because we can’t test them [villagers] we have to tell them to go to the pharmacy or health center to get tested. When they return with the result we give them ALU* [*a common anti-malarial medication*] *from the prescription they got from where they got tested, and most people do return to us and we give them treatment. When someone gets better s/he won’t say that WAJAs are not reliable, they believe that we are their doctors and that is the situation* (IDI, Male WAJA from Rufiji District).

WAJAs are not allowed to prescribe medicines without a proper diagnosis, and therefore, for example, ask their clients to get tested for malaria before receiving anti-malarials. Although WAJAs were supposed to be able to provide malaria testing through Rapid Diagnostic Test (RDT), during our observation period, their medical supplies were delayed, and most WAJAs had no RDT for diagnosis. In such cases, WAJAs referred their clients to the health facility for malaria tests and when the patients returned WAJA gave them medicine. Similarly, we found out that sometimes the health facility was also out of stock for essential medicines. The facility staff would refer their clients to private pharmacies. Instead of buying these medicines at the expensive private pharmacy, clients preferred to return to WAJA, where they could get the medicines for free. See Fig. 1 for more details on reverse referral. Whether WAJA diagnosed or recommended referrals to the health center before providing medication, community members perceived WAJAs’ position as doctor-like:

*Secondly, the difficulty for me is that people have begun seeing me like a doctor. I am talking about what is happening in my village. As a WAJA, I know that I must give medicines in the required dosage in order to avert the pressing diseases that affect children under five. But because [villagers] know you are a health care worker and you have some of the medicines, they think they don’t need to go anywhere else and every disease that one has will be treated by the WAJA.* (FGD, Male WAJA from Kilombero District)

Here the WAJA reflected on villagers’ attempt to elevate him as a doctor because of the WAJA’s access to medicine. However, the quote also shows the apprehension WAJAs have about such a label. None of the WAJAs we observed endorsed being called a doctor; they often highlighted that community members conflated access to medicine and adherence to dosage with a doctor-like identity.

The ability to prescribe medicine also enabled the WAJA’s professional role to deliver preventative services. And if WAJA were unable to provide medicine during their educative preventative house visits, their visit was often unwelcome. Due to challenges in the supply chain, WAJA often faced the challenge of needing to conduct their daily household visits to provide information without their curative services. WAJA’s reception in the house visits was very different during these times of curative shortages. A village supervisor in Rufiji observed:

*A certain belief has already been created in the client’s mind that if you are sick, the WAJA has medicine to cure you completely. And if a client visits the WAJA and he doesn’t have medicines, they question your [WAJA’s] purpose for visiting [the patient].* (IDI, Village Supervisor from Rufiji District)

Questioning in this context means that the patient described his visit to WAJA as a waste of time. Despite still being able to provide helpful information on nutrition and referrals, WAJA’s professional roles were unwelcome without the curative services. WAJAs are perceived to have failed in their professional role of providing medicine to their clients. Consequently, WAJA’s role as a health worker was challenged. While WAJAs service mandate was to serve mothers and children under five, everyone in the community required their services.

### WAJA’s local knowledge and efforts to provide family planning services

WAJA’s task of delivering family planning education and services to women and youth raised some moral and ethical questions from the community centering on issues of gender, reproduction and marital relations. WAJAs used their knowledge as locals to navigate this contentious terrain and provide personalized services to women who seek these services. Despite more than a decade of family planning education and services nationally, family planning remained a controversial topic in Tanzania including in Connect intervention areas.

One of the female WAJAs from Mlabani, Kilombero began her response to an interview question by providing insight into the contentious nature of family planning, and how it intersects with prevailing gender and patriarchal norms:

*In providing family planning services, we are always eager to involve the husbands because you can secretly give [pills to the wives] and later, the husbands finds the pills. [The wives to defend themselves] would say that the lady [WAJA] has given me the pills.* (IDI, Female WAJA from Kilombero District)

This female WAJA from Kilombero observed that it is best to approach the issue of family planning education and services by including the husbands. When a husband comes to know that his wife uses family planning or receives family planning education without his consent, she noted, the WAJA often becomes an object of anger and insults. Some husbands interpreted the use of family planning as an indication of their wives’ promiscuity. Some husband reasoned their wives could use pills to prevent pregnancy from extra-marital affairs.

The controversy surrounding family planning services was not confined to Kilombero area but extended to other Connect intervention areas such as Rufiji district. One of the female WAJAs interviewed from Mangwi Village in Rufiji District reported how she was confronted by an angry husband, whose wife did not tell him that she wished to use family planning services:

*I almost got in trouble but not entirely. There was a certain woman who wanted to start using family planning but she actually never met me … Some people directed her to my place and this news [that she is seeking family planning services] reached her husband. A fight broke out between the husband and the wife in their home. I had to call that woman to ask if there was a fight and she admitted that it happened. I told her if your husband doesn’t like it [family planning], I will have to come to your home to jointly inform you about the uses of family planning services and how it enables one to stop or resume pregnancy when one decides.* (IDI, Female WAJA from Rufiji District)

These two cases show that family planning remained a contentious subject, and women who wanted such services had to use them within constrained conditions. Males and particularly husbands were the main source of opposition for using family planning. Husbands’ anger was often directed toward their wives and the CHWs who were perceived to have offered these services. It happened several times that women enrolled in family planning services without their husband’s knowledge. In these cases, WAJAs approached their female clients as if they are “new” to the services:

*So if a woman has already secretly started the dose and her husband comes to know, he may think it’s her first time. There are women who start to use family planning [contraceptive pills] secretly by going to pharmacies and to the health centers without their husband’s knowledge—they go there early in the morning and return home un-noticed. So when you go to the likes of these clients you have to approach the whole situation as if you are persuading a new person to start family planning services. In fact, we are not allowed to give someone the pills; our work is to assist women who are already on the pills by continuing to support them.* (IDI, Female WAJA from Kilombero)

As locals, aware of the gender roles and patriarchal relations in the village, WAJA treated women who sought their services as new clients, meaning they respected their desire to use the services and minimize questions that probed into the women’s history of using contraceptives to make them feel more comfortable and less embarrassed.

In our study, we observed that WAJAs employed several strategies to enable new and existing users of family planning to continue utilizing the services. WAJAs met their clients away from their homes to give them advice or refill their pills. In certain cases, WAJAs reported that they recommended that their clients seek family planning services from bi-monthly outreach medical camps with local clinicians and nurses. These outreach programs are conducted away from their homes, where clients can speak with skilled clinicians and nurses about various family planning methods in relative privacy. A female WAJA from Lumemo, who has planned and participated in several outreach programs in her village, shared her experiences of medical camps and how they try to accommodate both new and existing clients:

*Husbands are usually comfortable allowing their wives to attend the outreach because it is a norm that mothers with children attend clinics for checking their infants’ weights, getting vaccinations and receiving safe motherhood education. When clients come to these outreach areas, in addition to the clinic duties, we counsel them about family planning services, and we connect them to nurses and clinicians who give them more detailed information and prescribe pills for them.* (IDI, Female WAJA from Kilombero District)

During the outreach, the WAJAs interviewed explained that they provided information to their new clients about family planning, but also meet with other clients with whom they had prior arrangements. Again, although these negotiations underpin the personalization of care, this is achieved at the cost of having to accommodate local patriarchal, gender and social norms.

WAJAs were also subjected to these gender and patriarchal roles with consequences to their professional work. There were differences in how community members received WAJAs and their services. In our observation, we found unmarried girls and married young women were more comfortable to speak to male WAJA about contraceptive use and refills than female WAJA. The general community stereotype was that women were gossipy and female WAJA’s professional role did not shield them from being perceived as gossipy. However, young males, both married and unmarried, were relatively comfortable in requesting condoms from both male and female WAJA. Male WAJA reported difficulty in speaking about family planning methods to older married female because they were being judged to be young and entering a domain of “women and sexual relations.” However, pregnant women and their relatives, in Kisawasawa for example, felt more comfortable to be escorted to the health centers at night by male WAJA than females. Villagers felt male WAJA could better protect them at night than female WAJA.

## Discussion

Using a mixture of IDI, FGD and ethnographic research, this article has examined the multiple, parallel roles of embedded CHWs (known as WAJAs). This examination demonstrates the complex ways in which the “professional” and “personal” identities of WAJAs interact, which has implications both for their work and their lives [37].

### WAJA’s participation in income generation enhances their professional identity

In our study, we found that WAJAs had been engaged in a wide range of income-generating activities prior to becoming paid health workers and that many continued these activities. Their continuation in these activities was related to the temporary nature of the project and the need to meet both family and community obligations. Involvement in income activities primarily as farmers but also as brick makers and motorcycle drivers enabled WAJA to utilize local social networks, reinforced their community relations and afforded them knowledge of the lives of the communities they served [1, 18]. This contrasted with health care workers who were recruited from different parts of Tanzania; spoke different languages; operated from a fixed-point and served for short-time. WAJAs local identities thus enhanced WAJAs professional identity by enabling them to form trusting and mutual relationships and better understand the challenges of providing care to diverse populations within the village, such as farmers, pastoralists, youth and women.

Our study also showed that personal and communal identities enhanced professional roles. By participating in the local economy such as farming, the WAJAs remained embedded in social, cultural and economic relationships that gave them a local identity and legitimacy. In this respect, our study supports the observation by Schneider et al. that showed CHWs’ position as “insiders” allowed them access to the community, converse in ways the villagers understood and therefore increased their efficacy to deliver services and feelings of being a professional health care provider. To be able to deliver their services, CHWs saw their knowledge and local network as an important means of doing their professional tasks [3]. In our study, WAJAs’ involvement in socio-economic activities such as farming, for example, kept them attuned to weather and infrastructural conditions as well as the emergence of new health challenges, such as outbreaks of cholera and waterborne diseases [32, 37]. This knowledge enabled CHWs to offer services that factored local realities and conditions, which in turn made them sought after by various health agencies including national health campaigns.

### Kinship ties: integrating project into local relations

Our findings on the importance of kinship relations with its implications to professional role is supported in other studies. In Kok et al. multi-country study, villagers trusted and felt comfortable to speak with community extension workers (HEWs) because they were “connected” to each other [19]. Program designers paid particular attention to the community ties of their applicants when recruiting HEW [19]. Other studies in Nigeria and South Africa also confirmed the centrality of kinship and their “insider” identity in positive and trusting relations built between CHW and their community [16, 19]. In our study, WAJAs were referred through kinship terms, which signaled their membership in the local community and their integral part in the existing social relationships. The use of kinship terms was so pervasive that the project staff such as the project supervisors also came to be referred through such terms. The supervisors, if a male, came to be known as Baba WAJA, meaning WAJA’s father and if she was a female, she came to be known as “Mama WAJA” which means WAJA’s mother. The use of kinship terms shows how personal and communal roles can interact with professional roles in creating trust and a sense of project’s ownership.

### Access to medicine and professional credibility

Several studies have shown how both access and lack of access to medicine affects CHWs roles and identities [16, 36]. A study by Ajayi et al. conducted in southwest Nigeria, concerning community perception of CHW providing home-based malaria management, found that communities positively evaluated CHWs provision of malarial drugs, even stating that they were more effective and more accommodating than health care workers [16]. In a multi-country study by Kok et al. detailing the benefits of CHWs as health intermediaries, the authors showed that in their case studies from Mozambique and Malawi community members valued their curative roles, which increased CHWs respect and recognition within the community [19]. Observation and interviews with WAJAs and their clients showed that access to medicine enhanced WAJAs’ social and professional status and gave them credibility as health providers. Our study thus supports other scholars’ findings that communities valued CHW’s curative roles [16, 38, 39].

Other studies have also shown the effects of irregular access to medicine and supplies on CHWs’ status [16, 36]. In the same study by Kok et al., the authors note that irregular medical supplies effected CHWs’ identity. When CHWs could not provide curative services because of either being overwhelmed with demand or lacking supplies, they felt stressed. At the same time, the community blamed and criticized them for not fulfilling their duties, which led some CHWs in Malawi to leave their homes [19]. Our conclusion accords with the findings of these studies: a lack or irregular supply of medicine does indeed have an inverse effect on WAJA’s personal, communal and professional status. When WAJA had access to medicine, they were judged as professional; when they lacked medicine, they were described as “mere youth” or as “of no use.”

### WAJAs as knowledge agents: offering family planning in constrained settings

WAJAs marshaled their socio-cultural and technical knowledge to provide culturally informed health services. Other studies have suggested that CHWs are knowledge agents, but have not adequately accounted for how “personal” identities and roles constitute a form of knowledge that CHWs draw upon [40, 41]. WAJA met their family planning clients in the streets or other convenient places, used respectful language, responded to questions and did follow-ups. They were able to do all this, it is clear, due to their community “embeddedness” [42]. But this “embeddedness” also accentuated several identities, some that aligned with the WAJAs’ project-assigned roles and others that conflict with them.

WAJAs approached family planning services by negotiating carefully between their personal and professional roles and larger socio-cultural forces. WAJAs were trained to offer family planning services to husband and wife together and publicly emphasize that using these services must be a joint decision. In a way, they were trying to change the discourse on family planning from being a women’s issue to a mutual decision involving couples [43, 44]. Such efforts have been widely documented in literature on family planning in Sub-Saharan Africa and Asia [45]. However, this official practice must be balanced against the right of women to choose whether and when to have children. When these two directives clashed, WAJA aided women desiring family planning in starting and continuing contraceptive services, even if the community and their husbands disapproved. Sensitive programs such as family planning tended to highlight WAJA’s status as youth and their gender. Clients who opposed family planning often accused WAJA for being either too young or, if they were males, of the wrong gender to speak about women’s reproductive issues [46]. Most of contention over family planning revolved arounds pills and come from husbands. As evidenced in the findings section above, WAJA frequently circumvented social and cultural structures such as gender, age and social status as well as opposition both from the community and from husbands in their effort to provide family planning services to women.

This circumvention was necessary because the WAJA’s position as “youths” in the village undermines their ability to actively confront gender and patriarchal norms that inhibit women’s ability to obtain family planning services [17]. In these cases, WAJA occupied conflicting roles: they were professional health workers with skills to meet clients’ needs, but also insiders with skills to avoid tensions between health workers and the community, arguably minimizing intra-household troubles that interfere with service initiation [28]. WAJA employed strategies such as referring their clients to health retreats, meeting them away from their home and suggesting family planning services that are less “visible” to women’s partners, such as injectable contraceptives. While such actions are not easily maneuverable at health facilities, these were some of the avenues available for providing individual family planning services which WAJAs were often uniquely well-positioned to provide them [46].

By using their socio-cultural knowledge to evade gender and social norms and provide family planning services to their clients, WAJAs both perpetuated existing norms and indirectly challenge them. As noted in other studies, family planning services and their promotion remains a contentious subject in many sub-Saharan and Asian countries [42, 47, 48]. By seeking the consent of husbands, parents, and elders, WAJAs perpetuated the patriarchal order of social and spousal relations. In these moments, we can say that WAJA were operating in their personal roles as youths who are socialized to respect elders and adhere to prevailing social norms. Yet their work might also be interpreted as an effort to subvert the traditional order, as in many cases they helped new and existing clients to use family planning services without their husband’s or society’s approval. Medical anthropologists have shown that women’s medical and health decisions are not a straightforward affair, and often entail moral and ethical ambiguities [49]. WAJA’s efforts to provide services to their clients expose these moral and ethical tensions.

### Gender and community norms and its implications to professional roles

Several studies have pointed out how gender and age, and socio-economic status have implications for CHW’s professional roles [3, 4, 20, 36]. Studies by Bhutta et al. and Haq et al. pointed to the importance of considering the amount of work CHWs do in relation to the effects of existing gender-based social cultural roles, and how these affected CHWs ability to provide care [4, 36]. Focusing on female’s mobility as a frame of analysis, Mumtaz et al. demonstrated how women’s cultural and social identities affected the number of visits female CHWs made, the places they could visit, and the quality of services. Factors like prior relationship between CHWs and the community, gender and community social norms emerged as important interface through which patients communicated with community extension workers (HEW) as well as how they were evaluated. Because the Health Extension Workers (HEW) work aligned with provision of maternal and child health such as clean and safe deliveries, post-natal care and family planning, the authors noted that patients preferred to speak to female HEW because they associated maternal and child matters to issues related to women [19].

The present study supplements these previous findings by showing how personal and professional identities interact with implications for professional and domestic life. In our study, we also found out that married and unmarried patients, depending on their age, preferred to discuss and access maternal and child health and family planning services from female CHWs, because it was culturally appropriate and related to what they viewed as women issues [17, 18]. However, younger women preferred to access and discuss contraceptive use with male WAJA because they felt less judged as promiscuous. With respect to young men aged 18–35, they were open to both female and male WAJA regarding family planning education and services such as condom refills.

## Conclusions and recommendations

CHW’s involvement in productive activities such as farming kept them in tune with their community’s social rhythms, economic patterns, and common health risks. One immediate implication of this finding is the importance of designing a realistic work schedule for CHWs that better accommodates their seasonal income-generating activities and family obligations. Because CHW’s kinship and communal bonds preceded each intervention, continued during it, and are renegotiated with the beginning of a new intervention, project staff should be aware that these identities and roles will both hinder and facilitate certain goals of the project. If a project is targeting supplies or medications only to a particular group, such as mothers and children under five, project leaders should be aware that CHWs will be pressured by the community to provide medications to other members within the social network. In other words, CHWs are members of the village and have a sense of obligation to share and serve despite the specific nature of their project mandate.

At present, the Tanzanian government is planning to scale the CHW program based on the Connect model [52]. It is necessary that the new national CHWs model retains some aspects of previous community engagement. “Embeddedness” within the socio-economic life of their villages transforms CHWs into powerful agents for preventative health care, but may also encourage deference to local power structures and norms. Our study found that village residency requirements, village selection and village oversight successfully created invested stakeholders in the project. This sense of community ownership of CHWs and their work was evident in the kinship names community members often used to refer to them.

CHWs also need supportive systems such as a reliable supply chain, supervision and mentorship, and a well-designed training package to be able to deliver services and maintain their status in the system (e.g. dispensaries) and above all in the communities they serve. All these resources are important in enabling them to gain positive identities, gain community acceptance, and provide the whole continuum of care from prevention to curative services and referrals. We suggest that program designers and the government should continue to pay CHWs for their services. If permanent arrangements are difficult to maintain, programs should be kept flexible and employers should be aware that CHWs may seek other sources of income to sustain their lives and fulfil their family obligations.

Our research focused on CHWs as a category. A further study is needed to explore how gender, marital, nuptial and social economic status of CHWs effect their personal and professional roles with implication to their work.

## Supplementary information

**Additional file 1.** IDI and FGD Questionnaires. The file contains questionnaires for conducting individual discussion interviews (IDIs) and focus group discussions (FGDs).

## Data Availability

The datasets used and/or analyzed during the current study are available from the corresponding author on reasonable request.

## Abbreviations

CHW: Community health worker
FGD: Focus group discussion
IDI: Individual interviews
MDG: Millennium Development Goals
RCT: Randomized controlled trial
WAJA: Wawezeshaji wa Afya ya Jamii
