# Supplementary material for: Who are CHWs? An ethnographic study of the multiple identities of community health workers in three rural Districts in Tanzania
Source: BMC Health Serv Res. 2019 Oct 21;19:712. doi: 10.1186/s12913-019-4563-6 (PMC6802175; doi:10.1186/s12913-019-4563-6)
Supplement: Supplementary file 1 — Additional file 1. IDI and FGD Questionnaires. The file contains questionnaires for conducting individual discussion interviews (IDIs) and focus group discussions (FGDs). [file 12913_2019_4563_MOESM1_ESM.docx]

Administrative data – *to be filled by researcher(s)*

DISTRICT:

IDI:

NAME OF THE VILLAGE:

DATE:

FACILITATOR NAME:

NOTE TAKER:

**Individual Discussion Interviews (IDI) Questions**

- Do you think there is any different between your workplan and the duties you’re actually performing?
  - What are those differences?

- In WAJA training, mothers and children were to be referred to local health centers, but this doesn’t always happen.
  - Can you tell me your views about this?
  - Are there particular periods when WAJA don’t use referrals?
  - Can you give examples when WAJA don’t use referrals?
  - Were you trained to give referrals?
  - In your village are there WAJA who don’t use referrals?

- How do you prepare your work timetable?
  - Do you involve your supervisors?
  - Can you tell me your weekly workplan?
  - What procedure did you use to prepare the plan and objectives?
  - What can you tell me about the development of your objectives and your workplan?
  - How do you make follow up on your objectives and workplan?
  - Who makes follow up on the developments of accomplishing those duties?
  - Who else follows up on your developments in accomplishing the objectives and completing your workplan?
  - Are government leaders involved?
  - Can you name them?
  - Can you tell me the success and the challenges which you face in accomplishing your objectives and workplan?
  - When you face the shortage of medical appliances how do you overcome this challenge?
  - When health care centers don’t have drugs, how do you handle the situation?
    - Where do you refer the patients then?

- Since you began working as WAJA in your community, have you seen changes in maternal and child health in your community?
  - What successes have you see?
  - How do you handle serious mother and child health cases?

- What is the working relationship between you and your supervisor?
  - When don’t you get enough assistance from your supervisor, what do you do?
  - Do you work with your colleagues to accomplishing your duties?

- What are the duties of the other stakeholders in helping WAJA in performing their duties?
  - How do you work with WAVI and other stakeholders?
    - How often and what specific tasks?

- How do you deal with the challenge of engaging men in family planning services?
  - What techniques do you use to encourage or to educate men?
  - When you go to the household, what techniques do you use to encourage both men and women?
  - Do stakeholders encourage men about the use of family planning?

- WAJA have been encouraged to work with different stakeholders, can you tell me the achievements from working with stakeholders?
  - Can you mention who the stakeholders are?
  - How do you cooperate with WAVI?
  - How did you cooperate with other stakeholders?
    - Which duties?

- What assistance do you need to accomplish your duties?
  - What type of assistance do you want?
  - Who are able to help you?

- Please describe your work relationship with your supervisor (that does not get paid to supervise your work)?
  - What aspects of the relationship work?
  - What aspects have had challenges?
  - What type of work have you done together?
  - What type of assistance do WAJA need from this supervisor?
  - What type of cooperation is needed?

- Is there good relationship between WAJA and the village leaders?
  - What are your suggestions in improving village supervision to support WAJA?
  - Should a village have a register? Why or why not?
  - Do some WAJA have a better relationship and/or cooperation with the village leaders than other WAJA in your village?
    - Why do you think this is?
  - What are the village government expectation towards WAJA?
  - How do they follow up on your WAJA duties?
  - What kind of assistance does WAJA need from the village government?
  - On the issue of attending mothers and children, what type of assistance do you want from the village government?
  - How do you cooperate with the village government in exchanging the information?
    - How do they use the information in making decisions?
    - What do WAJA need from village government to perform their duties?

- What kind of duties do you do together with your station supervisor?
  - What type of support do you need from your station supervisor?
  - What are your suggestions in improving the supervision from the station supervisor?
- What improvements have already been done?
- Do you depend on transportation or equipment from your station supervisor?

- How does the community support the WAJA?
  - Can you give examples?
  - What does WAJA need from the community to perform their duties?
  - How can community support be improved?

- When implementing WAJA in other areas, what are your suggestions to have it be successful?
  - Can you give examples?
  - How to better engage with village leaders?
  - What is needed from the community so that WAJA can perform the duties?
  - What suggestions do you have for the government? To the project?

Administrative data – *to be filled by researcher(s)*

DISTRICT:

FGD:

NAME OF THE VILLAGE

FACILITATOR NAME:

NOTE TAKER:

**Focus Group Discussion Interview (FGD) Questions**

- How did the WAJA program start in your village?
  - How did you plan to implement WAJA in your village?
  - Who can tell us the workplan which has been prepared and implemented in your village?
  - Can someone tell us the process which you prepared the workplan in your village?
- WAJA, what procedures did you use with your leaders or village government in preparing your daily time table?
  - Do you prepare your time table in cooperation with other supervisors?
  - Do you plan your time table in your villages and share it with other leaders?
  - Do you cooperate with the supervisor at the health center level?
  - How many meetings do you have with your supervisors?
  - Is there anyone with a different idea about the work arrangement or time table?

- What type of goals do you have?
  - What are short- and long-term goals?
  - What challenges do you face in your short- and long-term time tables?
  - What challenges do you have in the implementation of the planned work?
  - How do you solve them?

- What is your experience with supervisors?
  - How many supervisors do you have?
  - What are their duties?
  - How do you reach them?
  - Do you meet face-to-face or call them through phones?
  - How many times do you meet?
  - Give me an example of the reason why you meet.

- How is the situation of medical supplies for WAJA work?
  - Do you get all the medicines you need?
  - Apart from malaria medicines and condoms, are there other medicines that finish fast?
  - Can you give me examples when you were out of other medications or items?
  - Why do malaria medicines and condoms finish quickly?
  - Why do other supplies finish quickly?

- How did villagers respond to the shortage of medicines?
  - Can you give me an example of how villagers reacted?
  - What medicines or supplies were missing?
  - What did villagers say about the causes of the short supplies?
  - What do you think are the effect of the lack of medicines in your daily performance?
  - What do you think can be done in order to improve this situation or minimize the issue of lack of medicines and related materials?
  - Do you let your supervisors know about expired medicines?

- What type of cooperation is provided by the district?
  - Who do you work with at the district?
  - What are their duties and responsibilities?
  - When do you meet?
  - How many times?
  - What brings you together?
  - Do you get a response for your reports?

- Do health centers run out of medicines?
  - When does medicine run out?
    - Please give an example.
  - What kinds of medicine are stocked there?
  - What is the cause of medicine delays?

- How many WAJA are in your village?
  - How many are males?
  - How many are females?
  - Which cohorts are they from?
  - Do you work together?
    - Which issues do you work together?
  - Do you communicate with each other?
    - How?
    - Through phone or face-to-face meetings?

- Who do you report to?
  - Do you report to all the required levels?
  - Which are these levels?
  - How do you report?
  - Is there a requirement to report at a certain date or time?

- Do you give referrals?
  - Do you give referrals to the hospital?
  - What happens if the health centers don’t have certain medications?
  - Can anyone provide examples of referrals?
  - What challenges do you face when you are going to provide a referral from the households?
